# Initiate Danhong Injection before or after percutaneous coronary intervention for microvascular obstruction in ST-elevation myocardial infarction (DIRECTION): study protocol for a randomized controlled trial

**DOI:** 10.1186/s13063-019-3947-6

**Published:** 2020-01-08

**Authors:** Xiaoyu Zhang, Guihua Tian, Zhaofeng Shi, Yang Sun, Jiayuan Hu, Yin Jiang, Rui Zheng, Shiqi Chen, Chengyu Li, Xinyu Yang, Tianmai He, Songjie Han, Chi Zhang, Lijing Zhang, Yan Liu, Hongcai Shang, on behalf of the DIRECTION investigators

**Affiliations:** 1 Key Laboratory of Chinese Internal Medicine of Ministry of Education, Dongzhimen Hospital, Beijing University of Chinese Medicine, Beijing, 100700 China; 2 Institute of Basic Research in Clinical Medicine, China Academy of Chinese Medical Sciences, Beijing, 100700 China; 3 Department of Cardiology, Dongzhimen Hospital, Beijing University of Chinese Medicine, Beijing, 100700 China

**Keywords:** Danhong Injection, Percutaneous coronary intervention, Microvascular obstruction, ST-elevation myocardial infarction, Trial protocol

## Abstract

**Background:**

No treatment has convincingly been proven to be beneficial for microvascular obstruction (MVO) in patients with ST-elevation myocardial infarction (STEMI). Several studies have described the effects of Danhong Injection. However, evidence of a rigorously designed verification study is still lacking, and the intervention timing of Danhong Injection is uncertain.

**Methods:**

The DIRECTION study is a multicenter, prospective, randomized, evaluator-blind study. A total of 336 patients with STEMI receiving percutaneous coronary intervention (PCI) will be randomly assigned to conventional treatment, the preoperative Danhong Injection, or the postoperative Danhong Injection. The primary outcome is rate of ST-segment resolution (STR) ≥ 70% at 90 min after PCI. The secondary outcomes are the degree of STR, Thrombolysis in Myocardial Infarction (TIMI) flow grade, TIMI myocardial perfusion grade, left ventricular ejection fraction, N-terminal prohormone brain natriuretic peptide, high-sensitivity C-reactive protein, and infarct size expressed as area under the curve for cardiac troponin I (cTnI) and for creatine kinase MB. The major adverse cardiovascular events and hospital readmission events will be recorded. Health quality will be assessed with the 12-item Short Form Health Survey. The safety outcomes include bleeding events, adverse events, and abnormal changes in routine blood tests. Psychological status and dietary patterns will be evaluated using Hamilton Depression Rating Scale and Food Frequency Questionnaire as the relevant indicators.

**Discussion:**

This trial will evaluate the efficacy and safety of Danhong Injection, as well as its optimal timing of intervention to prevent MVO in patients with STEMI.

**Trial registration:**

Chinese Clinical Trial Registry, ChiCTR1900021440. Registered on February 21, 2019.

## Background

It is well known that myocardial infarction (MI) is associated with high mortality rates [1, 2]. According to the data of the China Cardiovascular Disease Report 2017 Summary, the mortality of acute myocardial infarction (AMI) in China is on the rise [3]. Prompt induction of complete and sustained infarct-related artery recanalization is paramount, and primary percutaneous coronary intervention (PCI) is currently the treatment of choice [4]. However, a significant proportion of patients still face low or no reperfusion at the myocardial tissue level, even though the infarcted blood vessels have been recanalized [5]. The incidence of slow or no reperfusion was 5–60%, depending on the study population, evaluation method, and time [6, 7]. The occurrence of slow or no reperfusion/microvascular obstruction (MVO) was associated with a lower ejection fraction, increased ventricular volumes and infarct size, and a greater risk of major adverse cardiac events (MACE), which severely affected the prognosis of patients who have had an MI.

MVO after PCI is a dynamic process involving multiple pathophysiological mechanisms [8]. The underlying pathological mechanisms are now known to include injury related to ischemia, reperfusion, endothelial dysfunction, and distal thromboembolism [6]. The complexity makes it not an ideal or standard treatment currently [9]. Different treatments have been tried, including thrombus aspiration and distal protective devices that can filter microemboli [9] and drug therapies such as vasodilators [10, 11], calcium channel blockers [12], nicorandil [13], cyclosporine A [14], platelet glycoprotein IIb/IIIa receptor antagonist [15], and glucagon-like peptide-1 analog [16]. There is currently experimental evidence that abciximab, adenosine, nicorandil, and nitrate are beneficial in reducing the incidence of no-reflow, but the clinical evidence is limited [17]. It is known that a traditional Chinese medicine (TCM) compound can interfere with the complicated pathological changes of the body for multitarget interactions [18]. In recent years, some clinical experimental studies have demonstrated the efficacy of TCM intervention in MVO and reperfusion injury after MI, revealing its unique advantages and potential application value in this field.

Danhong Injection is a TCM compound preparation extracted from *Salvia miltiorrhiza* and safflower [19]. It has the functions of promoting the blood circulation and clearing the vessels. The major active ingredients are tanshinone, salvianolic acid, safflower yellow pigment, phenol glycosides, and catechol. Danhong Injection has been widely applied in the treatment of coronary heart disease, angina pectoris, MI, pulmonary heart disease, and cerebral infarction since it was launched in the Chinese market in 2003. A recent systematic review showed that Danhong Injection significantly reduced the risk of death and recurrent angina, arrhythmia, and heart failure and improved left ventricular ejection fraction (LVEF) and reperfusion in patients with AMI [20]. Compared with PCI alone, Danhong Injection combined with PCI can improve vascular endothelial function, reduce inflammation, and prevent platelet aggregation, suggesting that it may act on multiple pathological steps, improve myocardial microcirculation, and reduce reperfusion injury [21]. Several small-sample clinical trials demonstrated that Danhong Injection can reduce the occurrence of coronary no-reflow (Thrombolysis in Myocardial Infarction [TIMI] blood flow grade < 3 or corrected TIMI frame count >40s) in patients with MI and can promote postoperative microcirculation recovery (ST-segment resolution [STR] ≥ 70% or 50%) [22–24]. However, limited research quality with the varieties of intervention timing and indicators makes it hard to come to a solid conclusion. More rigorously designed research and verification studies with a sufficient number of patients are warranted.

## Methods

### Purpose and study design

**Fig. 1:**
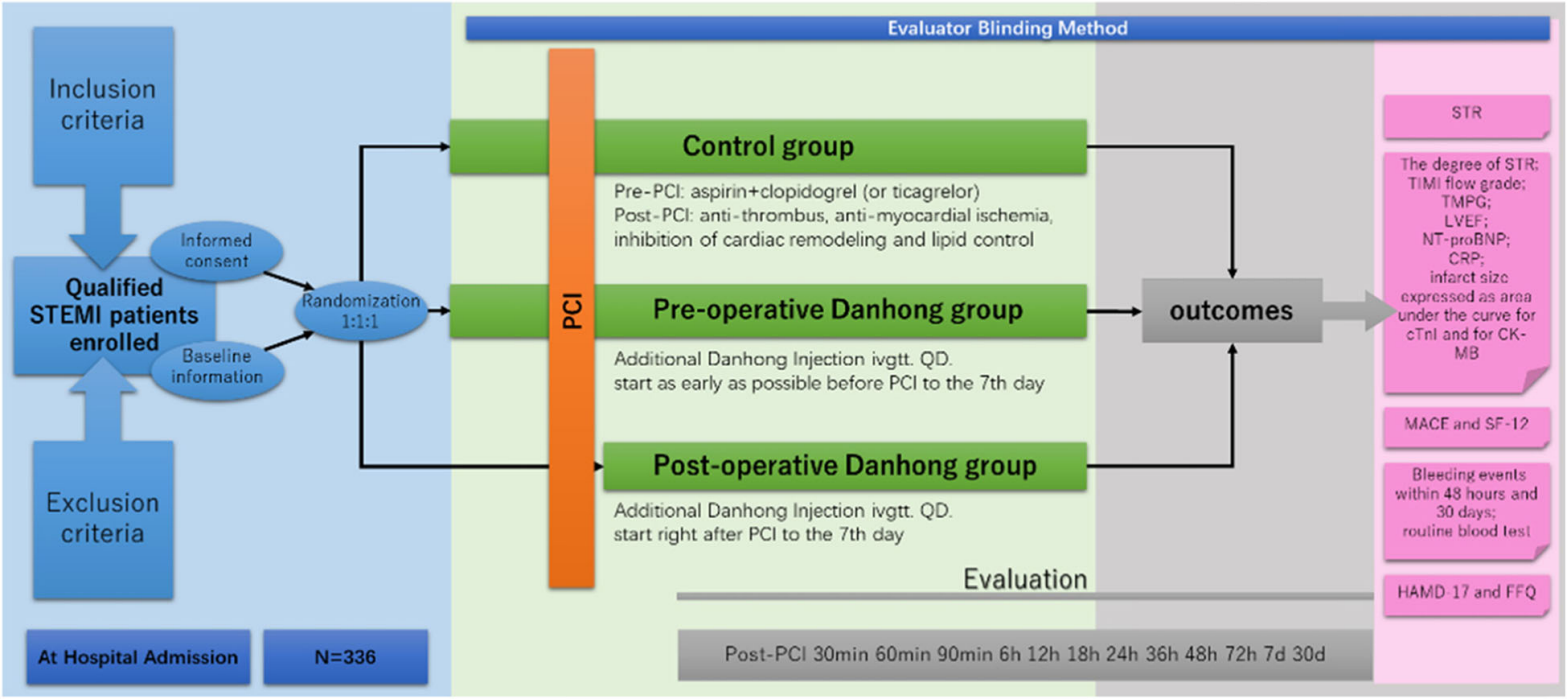
DIRECTION (Danhong Injection before or after percutaneous coronary intervention) trial protocol flow diagram

The purpose of this study is to assess whether additional Danhong Injection treatment started before or after PCI is superior to standard treatment alone for the prevention of MVO in patients with ST-elevation myocardial infarction (STEMI). The research is designed as a multicenter, prospective, stratified, block-randomized, evaluator-blind study being conducted within China from 1 May 2019 to 30 May 2020. A flowchart of the study is shown in Fig. 1. The enrollment of patients will take place from 1 May 2019 to 30 April 2020, and the observation period is 1 month. Central ethical approval has been confirmed by the Research Ethics Committee of Dongzhimen Hospital Affiliated to Beijing University of Chinese Medicine (ref. approval no. DZMEC-KY-2019-03). The trial was registered in the Chinese Clinical Trial Registry (ChiCTR1900021440). Reporting of the protocol follows the Standard Protocol Items: Recommendations for Interventional Trials (SPIRIT) statement (checklist uploaded as Additional file 1).

### Patient recruitment and eligibility

Patients will be screened for eligibility at hospital admission as soon as possible. Patients will be eligible if they meet the following criteria:

1. Diagnosed with STEMI according to the fourth edition of the 2018 European Society of Cardiology (ESC) General Definition of Myocardial Infarction [25] and the 2018 ESC Guidelines for Acute ST-Segment Elevation Myocardial Infarction [26]
2. Show symptoms of myocardial ischemia within 12 h
3. Have direct PCI surgery indications and accept stent placement in infarct vessels
4. Both men and women aged between 18 and 75 years
5. Agree to participate in the research and sign the informed consent form

The exclusion criteria are as follows:

1. Received thrombolytic therapy before PCI
2. Cardiogenic shock, heart rupture, or ventricular septal perforation
3. Cardiopulmonary resuscitation ≥20 min
4. Active hemorrhage or sensitive to hemorrhage
5. Target vessel received stent implantation
6. Having an implantable cardiac defibrillator or pacemaker
7. Combined with severe liver, kidney, or hematopoietic system diseases or malignant tumors
8. Allergic to the experimental drugs
9. Patients with major mental illnesses that make it difficult for them to cooperate
10. Known pregnancy or lactation
11. Acute pericarditis, subacute infective endocarditis, and/or aortic dissection
12. Left bundle branch block
13. Life expectancy ≤12 months
14. Participation in another clinical study with an investigational product or device during the last 30 days or during the study

### Intervention

Patients will receive aspirin 300 mg and clopidogrel 600 mg (or ticagrelor 180 mg) before PCI. Postoperative treatments will include antithrombotic medication (aspirin 100 mg daily with clopidogrel 75 mg daily or ticagrelor 90 mg twice daily), anti-myocardial ischemia (β-blockers, nitrates, calcium antagonists), inhibition of cardiac remodeling (angiotensin-converting enzyme inhibitor, angiotensin receptor blocker, aldosterone receptor antagonist), and lipid control (atorvastatin 20 mg daily). These are all serving as conventional treatment for patients with STEMI. Patients in the preoperative-Danhong group will additionally receive Danhong Injection treatment (40 ml with 250 ml of saline, intravenous drip infusion) as early as possible before PCI and continue to the seventh day, once per day. Patients in the postoperative-Danhong group will additionally receive Danhong Injection treatment (40 ml with 250 ml of saline, intravenous drip infusion) right after PCI and continue to the seventh day, once per day.

It should be noted that any Chinese medicine related to the treatment of coronary heart disease should not be taken orally, intravenously, or intracoronally during the study. According to the hospital centralized monitoring data [27], the following drugs should be avoided in combination with Danhong Injection: potassium magnesium aspartate, thymosin, celecoxib, and bisoprolol fumarate. The patients will stay in the hospital during the intervention period, which is convenient to monitor adherence to the intervention.

### Outcome

The primary outcome is the rate of STR ≥ 70% on surface electrocardiogram (ECG) at 90 min after PCI. The secondary outcomes are the degree of STR, TIMI flow grade, TIMI myocardial perfusion grade, LVEF, N-terminal prohormone brain natriuretic peptide, high-sensitivity C-reactive protein, and infarct size expressed as area under the curve for cardiac troponin I and for creatine kinase MB. The MACE (defined as a composite of nonfatal stroke, nonfatal MI, target lesion revascularization, urgent stent thrombosis, and all-cause death) will be recorded, as well as the hospital readmissions for heart failure or cardiac angina. Health quality will be assessed by using the 12-item Short Form Health Survey [28].

The safety outcomes include bleeding events measured with Platelet Inhibition and Patient Outcomes (PLATO) criteria within 48 h and 30 days, as well as adverse events (AEs) during the study period. Abnormal change in routine blood test results will also be monitored during the study. In addition, psychologic status and dietary patterns of patients will be collected as relevant indicators using the Hamilton Depression Scale [29] and the Food Frequency Questionnaire [30]. A study schedule and evaluation of outcomes are provided in Table 1. Centralized, blinded reviews of angiographic data and ECG recordings will be conducted by two physicians, respectively.

**Table 1:** Outcome evaluation time points

Outcomes	Baseline	Preoperation	Postoperation (immediately)	30 min	60 min	90 min	6 h	12 h	18 h	24 h	36 h	48 h	72 h	7 d	30 d
Subject characteristics	•	–													
STR	•	–		•			–			•	–			•	–
TIMI flow grade TMPG	–	•		–											
LVEF	–									•	–			•	
cTnI, CK-MB	•	–					•							–	
NT-proBNP	•	–								•	–	•	–	•	
hs-CRP	•	–								•	–		•		
MACE	–														•
Hospital readmission	–														•
SF-12	•	–													•
Bleeding events	–											•	–		•
Adverse events	•														
Routine blood test	•	–													•
HAMD-17	•	–												•	
FFQ	•	–													

Abbreviations: *CK-MB* Creatine kinase-MB, *cTnI* Cardiac troponin I, *FFQ* Food Frequency Questionnaire, *HAMD-17* Hamilton Depression Scale-17, *hs-CRP* High-sensitivity C-reactive protein, *LVEF* Left ventricular ejection fraction, *MACE* Major adverse cardiovascular event, *NT-proBNP* N-terminal prohormone brain natriuretic peptide, *SF-12* 12-item Short Form Survey, *STR* ST-segment restoration, *TIMI* Thrombolysis in Myocardial Infarction, *TMPG* Thrombolysis in Myocardial Infarction myocardial perfusion grade

*Note*: • = essential item

### Sample size

Based on the results of the previous investigation [31–33], a sample size of 336 participants was estimated to provide 80% power to detect an absolute difference of 20.2% between the combined Danhong group and control in the rate of STR ≥ 70% measured at 90 min after PCI at a two-sided significance level of 0.05, assuming an STR ≥ 70% rate of roughly 50% for the control group and 20% loss to follow-up.

### Randomization

In each center, the enrolled patients will be randomly assigned to the control group of conventional treatment, the preoperative-Danhong group, or the postoperative-Danhong group, allocated by the central randomized management system according to a ratio of 1:1:1. The randomization sequence will be generated in varying block sizes and stratified by center. After informed consent is obtained, the researcher will log into the central randomized management system of the network and choose the center as the stratified factor. The central randomized management system will generate a unique identification code and random number for each patient, which can be used to represent the identity of the patient and indicate the therapeutic intervention.

### Data collection and management

The data will be managed according to standard operating procedures (SOPs). The data from the paper case report form (CRF) will be entered and stored in the electronic data capture system. A specialized quality inspector will regularly review CRFs and monitor the data. Monitoring results should be presented to the principal investigator in each center, who is responsible for the accuracy, completeness, and timeliness of the data recorded. After blind review and confirmation that the established database is correct, the data will be locked, and no changes will be permitted.

### Statistical analysis methods

The null hypothesis is that the rate of STR ≥ 70% at 90 min after PCI will be the same for the combined Danhong group and the control group. The primary outcome analysis will use the Cochran-Mantel-Haenszel test to compare the rate of STR ≥ 70% at 90 min after PCI between the combined Danhong groups and the control group. If the result of this analysis is significant, hierarchical testing will be used to compare the preoperative-Danhong group with the control group, the postoperative-Danhong group with the control group, and the preoperative-Danhong group with the postoperative-Danhong group.

For other categorical variables, comparisons between treatment groups will be done using Fisher’s exact test or the chi-square test as appropriate. Continuous variables will be compared using the *t* test or Wilcoxon rank-sum test as appropriate. Chi-square or Fisher’s exact tests will be used to compare the frequency of AEs between groups.

The intention-to-treat population, including all randomized patients, will be used for all efficacy and safety analyses. All analyses will be performed using SAS version 9.4 software (SAS Institute Inc), with a two-sided *P* value less than 0.05 considered significant. No interim efficacy analysis will be performed.

### Quality management

Study physicians and evaluators will be trained in SOPs in advance. The ECG and angiographic images from a subcenter should be sent to the data center for unified blinding evaluation to obtain high-quality research data. The accuracy, reliability, and abnormal judgment criteria of laboratory tests should be unified in each research center.

### Adverse events

The trial will be monitored by an independent data monitoring committee (DMC) comprising experts in cardiovascular disease, clinical trial methodology, statistics, and ethics. The DMC assigns the severity of AEs as mild, moderate, severe, or serious AEs. Any AEs that occur during the study process should be recorded in the AEs form, including the time, severity, and duration of the AEs; the measures adopted; and the outcomes. These AEs will be addressed appropriately, and the treatment measures and results will be recorded. The subjects with AEs will be followed up until they are properly recovered or their condition is stable.

The relationships of AEs to the research medication are assessed by the DMC. The causal judgment indicators include whether the administration time and the suspected AEs exhibit a reasonable relationship; whether the suspected AEs fulfill the criteria for the typical reactions of the drug; whether the AEs can be explained by the effects of the combined drug, patient’s clinical condition, or other therapies; whether the suspected adverse reactions disappear or are mitigated after discontinuation of the drug; and whether the same reaction recurred after repetitive administration of the research medication.

### Ethical plan

This clinical trial protocol follows the Helsinki declaration (October 2008 version; 49th General Assembly of the World Medical Association, Somerset West, Republic of South Africa) and Chinese clinical trials regulations. The protocol can be implemented only after the approval of the ethics committee. When the protocol is revised, the trial process must be approved by the ethics committee, and the informed consent needs to be signed again. Insurance will be provided for subjects participating in the study. Only the researchers and monitors can view the personal medical records of participants anonymously for protection of their privacy. All participants will be informed about the nature of the trial, its aims, expected advantages, and possible risks. All eligible participants will provide written informed consent, and in case of patient incapacity, the legally authorized representative will provide it. The protocol and the results of the present study will be published in peer-reviewed journals or scientific conference presentations according to the guidelines of the Standard Protocol Items: Recommendations for Interventional Trials (SPIRIT) and Consolidated Standards of Reporting Trials (CONSORT) statements.

### Discontinuation and suspension conditions

The purpose of the suspension is to protect the rights of the participants, to ensure the quality of the study, and to avoid unnecessary economic losses. If the clinical trial needs to be suspended, the research group and regulatory authorities should be notified in time, and they will decide whether to continue the present study. The discontinuity conditions are as follows: First, the clinical trial program has major errors, or although the trialed program is reasonable, there are serious deviations during the implementation and the efficacy cannot further be evaluated; second, the trial sponsor requires the suspension; and third, the administrative department aborts the trial. It should be noted that all CRFs should be retained for future investigation.

## Discussion

Over the years, efforts have been made to detect an effective strategy to prevent and alleviate MVO in time windows of STEMI treatment, but currently no treatment has convincingly been proven to be beneficial in a multicenter controlled randomized trial with clinical outcomes [17]. And it is uncertain that whether early drug intervention could improve coronary reperfusion in patients with STEMI for primary PCI [34]. Our primary purpose is to evaluate whether additional Danhong Injection use is superior to conventional treatment alone for the prevention of MVO in patients with STEMI undergoing PCI. Furthermore, we hope to find the optimal timing of Danhong Injection intervention, which would be helpful to improve the clinical effect of TCM medications and to guide rational drug use.

Several clinical trials have been carried out to evaluate the effectiveness of TCM for the prevention and treatment of no-reflow in recent years. A randomized, double-blind, placebo-controlled, multicenter clinical trial evaluated the no-reflow protective and long-term effects of Tongxinluo capsules for AMI. The trial researchers observed that the reduction of no-reflow incidence was time-dependent in the Tongxinluo group with statistical significance at 24-h reperfusion compared with the control group. The infarction area determined by single-photon emission computed tomography was reduced on day 7 and day 180 after STEMI in the Tongxinluo group compared with the control group [35]. Compared with oral preparation, Danhong Injection has an advantage in taking rapid effect in the short time window of myocardial reperfusion. Systematic reviews have found that Danhong Injection is associated with improvement of reperfusion and LVEF, as well as reduction of MACE in acute coronary syndrome when combined with PCI [20, 21].

STR based on ECG is the simplest clinical evidence of effective myocardial reperfusion in myocardial cells and has been confirmed to have predictive value [17, 36]. We are adopting ECG to assess the MVO as the primary outcome, considering it is noninvasive, which makes it easy to observe the reperfusion trends between the three groups. Also, blinded reviews of ECG recordings and angiographic data will be conducted by two physicians independently to avoid potential evaluation bias.

This trial has the following advantages:

1. It will be the first and largest well-designed, multicenter randomized controlled trial with rigorous quality control to evaluate the efficacy and safety of Danhong Injection for MVO prevention in patients with STEMI.
2. No previous research has evaluated the optimal intervention timing of TCM medications during the perioperative period of PCI, and our trial will fill that gap.

As for the limitations, a placebo-controlled and double-blind method will not be applied in this trial, which may bias the results. We choose to use a blank control group mainly because the preoperative-Danhong group and postoperative-Danhong group need to share a common control for the purpose of identifying the optimal timing of intervention. It is not possible to start placebo use at two different time points in one group, not to mention that it is hard to make the Danhong Injection placebo with the same color. However, this study applies an evaluator-blind method to reduce evaluation bias. In addition, the intensive evaluation time points bring difficulty to trial implementation. We will strengthen the SOP training and monitoring to ensure the soundness of the process.

This study will be the first and largest well-designed, multicenter randomized controlled trial with rigorous quality control to evaluate the efficacy and safety of Danhong Injection, as well as its optimal timing of intervention, to prevent MVO in patients with STEMI. The results of this trial will provide valuable clinical evidence for recommendations on the management of the disease and clues to the underlying mechanisms.

### Trial status

The study is currently in the process of recruiting participants. Recruitment of participants commenced on 30 June 2019 and will be completed in May 2020.

## Supplementary information

**Additional file 1.** SPIRIT 2013 checklist.

## Data Availability

The datasets generated and/or analyzed during the current study are not publicly available, owing to the protection of privacy for patients, but they are available from the corresponding author on reasonable request.

## Abbreviations

AE: Adverse event
AMI: Acute myocardial infarction
CK-MB: Creatine kinase MB
CMH: Cochran-Mantel-Haenszel
CONSORT: Consolidated Standards of Reporting Trials
CRF: Case report form
cTnI: Cardiac troponin I
DMC: Data monitoring committee
ESC: European Society of Cardiology
FFQ: Food Frequency Questionnaire
HAMD-17: Hamilton Depression Scale-17
hs-CRP: High-sensitivity C-reactive protein
LVEF: Left ventricular ejection fraction
MACE: Major adverse cardiovascular event
MI: Myocardial infarction
MVO: Microvascular obstruction
NT-proBNP: N-terminal prohormone brain natriuretic peptide
PCI: Percutaneous coronary intervention
SF-12: 12-item Short Form Health Survey
SOP: Standard operating procedure
SPIRIT: Standard Protocol Items: Recommendations for Interventional Trials
STEMI: ST-elevation myocardial infarction
STR: ST-segment resolution
TCM: Traditional Chinese medicine
TIMI: Thrombolysis in Myocardial Infarction
TMPG: Thrombolysis in Myocardial Infarction myocardial perfusion grade
